# YhjC is a novel transcriptional regulator required for Shigella flexneri virulence

**DOI:** 10.1080/21505594.2021.1936767

**Published:** 2021-06-21

**Authors:** Wanwu Li, Lingyan Jiang, Xiaoqian Liu, Rui Guo, Shuai Ma, Jingting Wang, Shuangshuang Ma, Shujie Li, Huiying Li

**Affiliations:** a TEDA Institute of Biological Sciences and Biotechnology, The Key Laboratory of Molecular Microbiology and Technology, Ministry of Education, Nankai University, Tianjin 300457, China; b Shandong Center for Food and Drug Evaluation & Certification, Jinan, China; c Department of Clinical Laboratory Medicine, The First Affiliated Hospital of Shandong First Medical University & Shandong Provincial Qianfoshan Hospital, Shandong Medicine and Health Key Laboratory of Laboratory Medicine, Jinan 250014, China; d School of Basic Medicine, Shandong First Medical University & Shandong Academy of Medical Sciences, Jinan 250062, China

**Keywords:** *Shigella flexneri*, yhjc, regulator, *virF*, virulence

## Abstract

*Shigella* is an intracellular pathogen that primarily infects the human colon and causes shigellosis. *Shigella* virulence relies largely on the type III secretion system (T3SS) and secreted effectors. VirF, the master *Shigella* virulence regulator, is essential for the expression of T3SS-related genes. In this study, we found that YhjC, a LysR-type transcriptional regulator, is required for *Shigella* virulence through activating the transcription of *virF*. Pathogenicity of the *yhjC* mutant, including colonization in the colons of guinea pigs as well as its ability for host cell adhesion and invasion, was significantly lowered. Expression levels of *virF* and nearly all VirF-dependent genes were downregulated by *yhjC* deletion, indicating that YhjC can activate *virF* transcription. Electrophoretic mobility shift assay analysis demonstrated that YhjC could bind directly to the *virF* promoter region. Therefore, YhjC is a novel virulence regulator that positively regulates the *virF* expression and promotes *Shigella* virulence. Additionally, genome-wide expression analysis identified the presence of other genes in the large virulence plasmid and a genome exhibiting differential expression in response to *yhjC* deletion, with 169 downregulated and 99 upregulated genes, indicating that YhjC also functioned as a global regulatory factor.

## Introduction

*Shigella* is a Gram-negative enteropathogenic bacterium that causes shigellosis, a common disease that reportedly occurs worldwide, with higher incidence in developing countries. The infection is characterized by the occurrence of diarrhea, often with blood or mucus present in the feces [1]. *Shigella* gains entry into the human colonic lumen via the fecal-oral route and primarily targets the M cells of the colon, resulting in cell death, acute inflammation, and tissue edema. The released bacteria further enter the colonic enterocytes from the basolateral side [2]. Virulence proteins directly involved in the entry and dissemination of *Shigella* in host cells are encoded by a large, approximately 200-kb virulence plasmid, containing a 31-kb region (designated “entry region”) encoding the Type III secretion system (T3SS) [1,3]. VirF, a DNA-binding activator belonging to the family of AraC-type transcription regulators, is considered the master regulator that activates the expression of T3SS and secreted virulence proteins by regulating at least two primary *Shigella* virulence factors, including *virB* and *icsA* [4,5]. VirB can directly enable the activation of the transcription of genes encoding the entry region [6,7]. Therefore, VirF is vital for *Shigella* invasion and colonization of the colon.

Despite the critical invasion processes and important virulence effectors reported in the existing literature, much remains unknown about the regulators and the corresponding regulatory mechanisms underlying *Shigella* pathogenesis. For instance, only a few regulators, including Fis, CpxA/R, and IHF, which can acrivate *virF* expression in response to environmental stimuli, have been investigated [8–10]. However, the existence of other factors that act as *virF* regulators still remains to be investigated. In this study, we found that a new LysR-type transcriptional regulator (LTTR), YhjC, was required for *Shigella* virulence. The results showed that YhjC could promote *Shigella* pathogenicity by activating *virF* transcription, indicating that YhjC functioned as a new virulence regulator. Furthermore, transcriptome mapping results revealed that more than 200 genes were regulated by YhjC, indicating that it also played roles as a global regulator.

Hence, our study not only identified YhjC, a previously unrecognized regulator in the LysR family that plays a role in virulence and global regulation of transcription in *Shigella*, but also provided novel insights into *virF* expression and the regulation of *Shigella* pathogenesis.

## Results

### *The* yhjC *mutant exhibits limited efficiency in invading guinea pigs colon*

**Figure 1.**
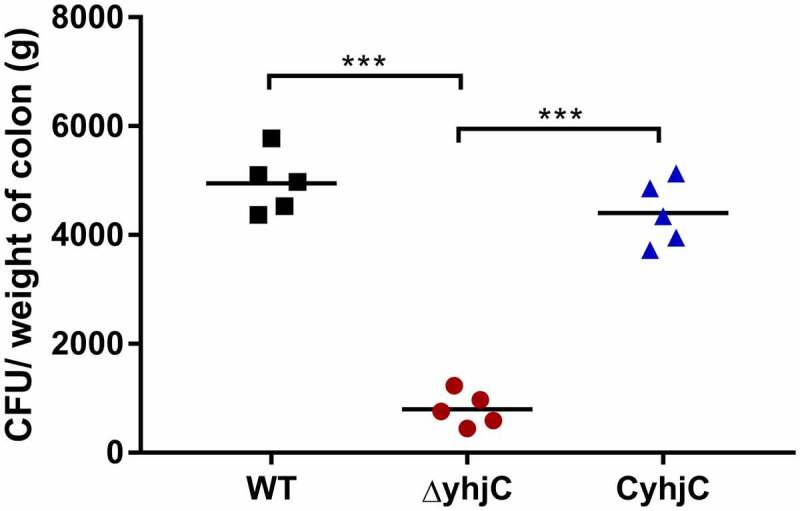
Intrarectal infection of Guinea pigs with the wild type (WT) and Δ*yhjC* strains. The number of colony-forming units (CFUs) of the WT strains colonizing colon tissues was significantly higher than those of Δ*yhjC*, and the complementation of *yhjC* with Δ*yhjC* restored the ability of mutant *Shigella* strains to colonize the colon tissues to levels observed with the WT strain. 5 guinea pigs per group were used. Data were obtained from two separate experiments and analyzed using unpaired student’s *t*-test (*p < 0.05; **p < 0.01; ***p < 0.001)

*Shigella* invades the colonic mucosa, leading to the occurrence of acute inflammatory colitis, dysentery, and intestinal hemorrhage, which destroy the intestinal epithelium [11]. We previously found that *yhjC* expression was activated during *Shigella* infection; thus, we investigated whether YhjC was necessary for *Shigella* pathogenicity. We infected guinea pig colon tissues with the wild type (WT) *Shigella flexneri* (*S. flexneri*) 5a strain M90T and the *yhjC* mutant (Δ*yhjC*) strain. Colony-forming units (CFU) of the WT bacteria were 6.20 times more than those of the *yhjC* mutant in the infected colon (Figure 1), indicating that YhjC was a crucial virulence regulator during the establishment of in vivo *Shigella* infection. The CFU of complementation was similar to that of WT.

### *The* yhjC *mutant exhibits limited efficiency in adhering to and invading host cells*

**Figure 2.**
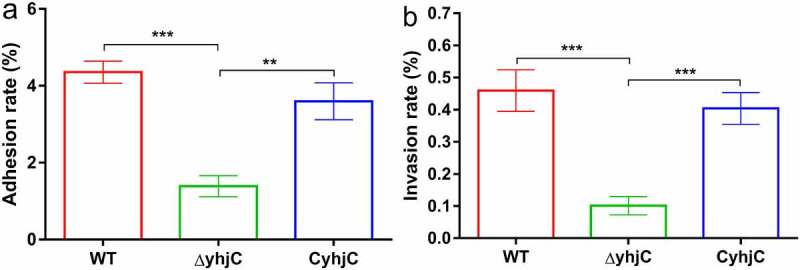
Analysis of the ability of the wild type (WT), Δ*yhjC*, and complemented *Shigella* strains to adhere to and invade HeLa cells. Both adhesion and invasion abilities were attenuated in Δ*yhjC* mutants compared with those observed in the WT, and the complemented strain showed adhesion and invasion abilities similar to the WT. (a) Analysis of adherence ability; (b) analysis of invasion ability. Data were generated from three independent experiments and have been presented as mean ± SD. *p*-values were determined using unpaired Student’s *t*-test (*p < 0.05; **p < 0.01; ***p < 0.001)

The reduction in the pathogenicity of Δ*yhjC* may be attributed to the attenuated capacity of the bacteria to infect host cells. To validate this hypothesis, we investigated the ability of the WT and Δ*yhjC* bacteria to adhere to and invade HeLa cells. The ability of the *yhjC* mutant to adhere to HeLa cells was approximately 3.14-fold lower than that of the WT strain, and the complement strain (C*yhjC*) exhibited significantly higher adherence ability compared to the *yhjC* mutant (Figure 2a). The ability of the *yhjC* mutant to invade HeLa cells was not as robust as that of the WT, and decreased by 78.01%, although this invasion ability was regained in the C*yhjC* strain (Figure 2b). These results indicate that *yhjC* deletion causes pathogen deficiency in terms of host cell adherence and invasion, and YhjC is therefore essential for M90T invasion.

### yhjC *deletion decreases the Congo red dye-binding ability of* Shigella

**Figure 3.**
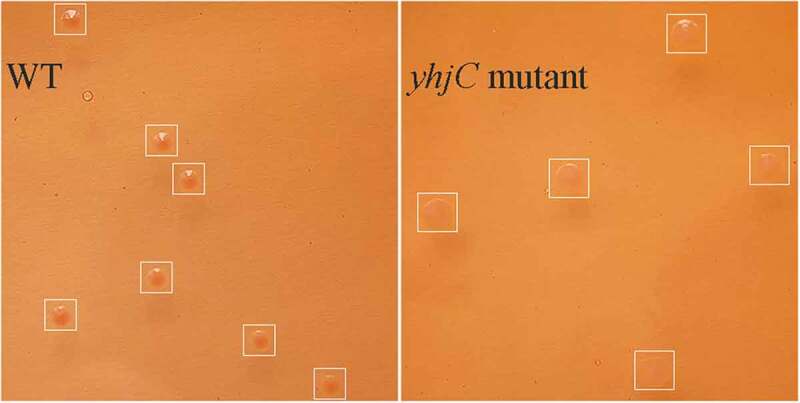
Comparison of the Congo red dye-binding ability of the wild type (WT) and Δ*yhjC* strains. The WT bacterial colony exhibits increased intensity of red coloration than that observed with Δ*yhjC*, thereby proving that WT strains are more virulent than *yhjC.*

Based on the results mentioned above, as T3SS and the secreted virulence proteins contribute to *Shigella* infection and invasiveness, we speculated that YhjC might affect T3SS activity. The ability of *Shigella* to bind Congo red dye was positively associated with T3SS activity and the virulence of this pathogen [12,13]. WT bacterial colonies exhibited red coloration with higher intensity than the *yhjC* mutant colonies (Figure 3). The OD_498_/OD_600_ of WT colonies was 0.290, which was significantly higher than that of *yhjC* mutant colonies (0.097), indicating that T3SS activity and virulence of WT were higher than those demonstrated by the *yhjC* mutant. Thus, the results imply that YhjC is essential for T3SS activity, which is activated by VirF.

### *YhjC promotes* Shigella *virulence by activating* virF

**Figure 4.**
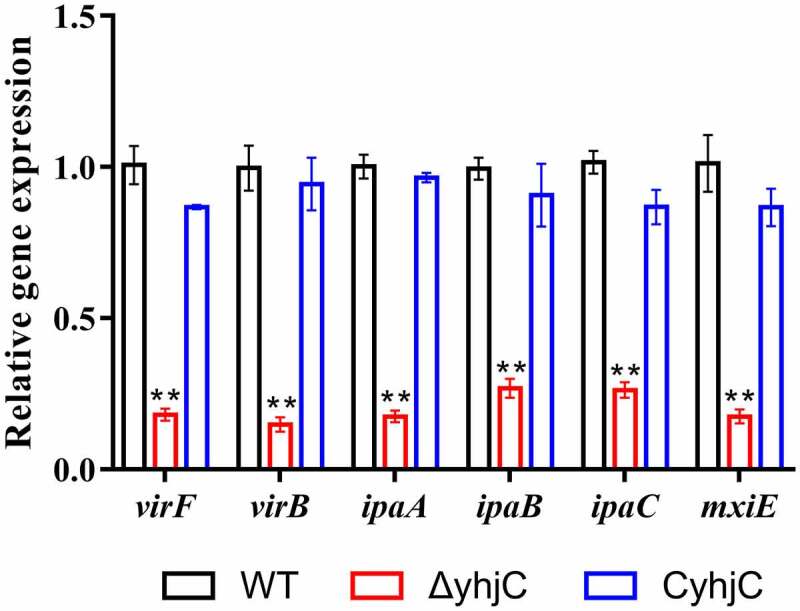
*yhjC* deletion downregulates the expression of the main virulence genes of *Shigella*. The expression levels of *virF, virB, ipaA, ipaB, ipaC*, and *mxiE* were significantly downregulated in Δ*yhjC*. Data were generated from triplicate experiments and have been presented as mean ± SD. *p*-values were determined by using unpaired Student’s *t*-test (*p < 0.05; **p < 0.01; ***p < 0.001)

Based on the phenotypic results, we further speculated that YhjC promoted *Shigella* virulence by controlling the expression of virulence-related genes such as T3SS-related genes, and genes encoding the virulence proteins *virB* and *virF*. As the identity of the gene regulated by YhjC was unknown, we selected several invasion-related genes (*ipaA, ipaB*, and *ipaC*) and virulence regulators (*virF, mxiE*, and *virB*) and compared their expression in the *yhjC* mutant and the WT strain using quantitative real-time PCR (qRT-PCR). Expression levels of all identified genes were downregulated in response to *yhjC* deletion (Figure 4). As the identified genes were regulated by VirF, we concluded that YhjC promote *Shigella* virulence by activating *virF*.

### *YhjC binds directly to the promoter region of* virF

**Figure 5.**
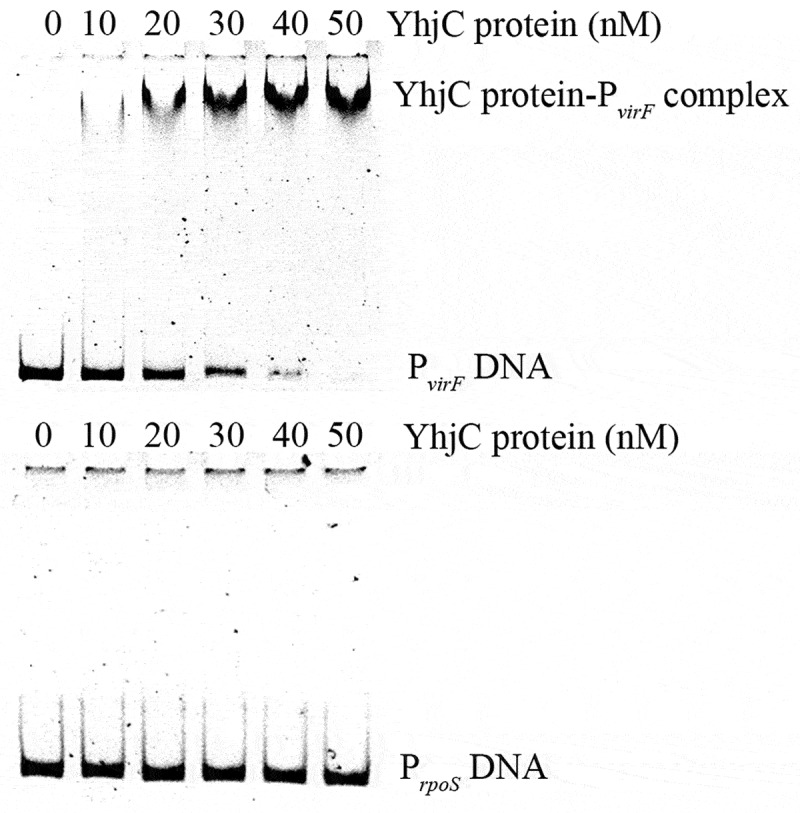
Analysis of the interaction between the purified YhjC-His_6_ protein and the *virF* promoter DNA using EMSA. A quantity of 50 ng of DNA and a concentration ranging from 0–50 nM of the purified YhjC-His_6_ protein were used in each reaction. Less promoter *virF* fragments exhibited migration with increasing concentrations of the YhjC protein. The DNA of the *rpoS* promoter was used as a negative control and no binding was observed

To determine the way of YhjC activating *virF* expression, we explored the interaction between the purified YhjC-His_6_ protein and *virF* promoter DNA using electrophoretic mobility shift assay (EMSA). We observed that a reduced quantity of the *virF* promoter DNA (P*_virF_* DNA) exhibited migration under increasing concentrations of the YhjC protein, whereas no movement retardation was observed for the *rpoS* promoter, which was used as the negative control DNA (Figure 5). Therefore, we confirmed that YhjC bound directly to the *virF* promoter.

### RNA sequencing analysis

#### Transcriptional profile

**Figure 6.**
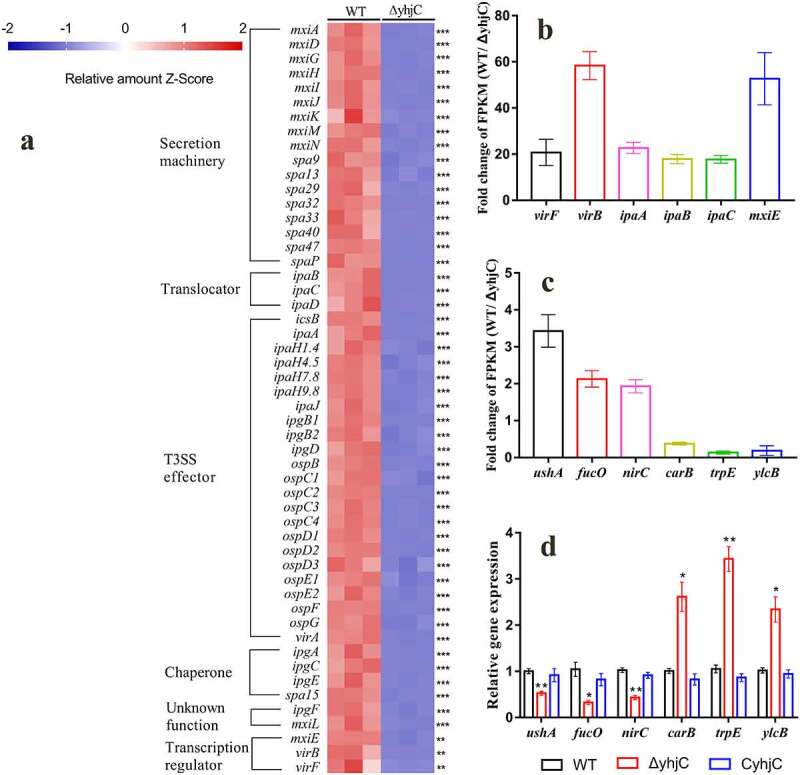
RNA sequencing analysis. (a) Transcriptional profiles of T3SS-related genes in the wild type and Δ*yhjC Shigella* strains. Expression levels of almost all T3SS-related genes were downregulated following *yhjC* deletion. (b, c) Fold-change in the FPKM of representative differentially expressed genes (DEGs). The fold change in representative DEGs encoding virulence genes is much higher than that observed in non-virulence genes. (d) Verification of the RNA sequencing data through qRT-PCR analysis of non-virulence genes. Changes in the expression of the non-virulence genes are consistent with those of the RNA sequencing data. Data were generated from triplicate experiments. *p*-values were determined by using multiple *t*-test and unpaired Student’s *t*-test (*p < 0.05; **p < 0.01; ***p < 0.001)

LTTRs are ubiquitous proteins that regulate the expression of a variety of genes [14]. We further investigated the global regulatory function of YhjC in *Shigella* by performing sequencing analysis of the RNA samples obtained from the WT and *yhjC* mutant strains. A total of 15,003,465 and 16,435,097 reads were obtained upon analysis of the WT and *yhjC* mutants, respectively, with 98.92% and 98.73% of the reads mapping to the reference genome. 268 genes were differentially expressed between the *yhjC* mutant and the WT strain, including 169 downregulated genes (Figure 6a, Table S2) and 99 upregulated genes (Table S3). Gene ontology (GO) enrichment analysis and Kyoto Encyclopedia of Genes and Genomes (KEGG) pathway enrichment analyses were performed. Please check the Supplementary Material (Figure S2 and Figure S3) for the results of these analyses.

#### yhjC *deletion downregulates the expression of virulence-related genes*

RNA sequencing data showed that the expression levels of almost all virulence-related genes were downregulated in the *yhjC* mutant compared with the WT strain (Figure 6a and 6b), and this downregulated expression showed appreciable correlation with the qRT-PCR results (Figure 4). The expression of *virF* was downregulated 20.68-fold in the *yhjC* mutant. Correspondingly, expression levels of *virB* and *icsA*, which are directly regulated by VirF, were downregulated by 59.57-fold and 4.90-fold, respectively. VirB can activate the transcription of multiple virulence genes, including genes encoding the T3SS secretion machinery (Mxis and Spas), translocators (Ipas), chaperones (Ipgs), “first-set” effectors (IcsB, OspC2-4, OspD1/2, etc), MxiE, and the outer membrane protease (IcsP) specific for the actin-based motility protein IcsA [15,16]. Consistent with *virB* downregulation, expression levels of the VirB-regulated genes were also downregulated by 4–71-fold. The transcription of genes encoding the “second-set” T3SS effectors is controlled by the MxiE transcriptional activator, with the IpgC chaperone acting as a coactivator [1,17]. Expression levels of the genes, including *virA, ipaHs, ospB, ospC1, ospD3, ospE1/2, ospF*, and *ospG*, were downregulated 2–16-fold.

#### Validation of RNA sequencing results

To validate the RNA sequencing results, qRT-PCR analysis was performed using six additional randomly selected genes, including *ushA, fucO, nirC, carB, trpE*, and *ylcB*. The mRNA levels of these six genes were consistent with the RNA sequencing results (Figure 6c and 6d), thus validating the reliability of the RNA sequencing data.

### *The effect of environmental conditions on* yhjC *expression*

**Figure 7.**
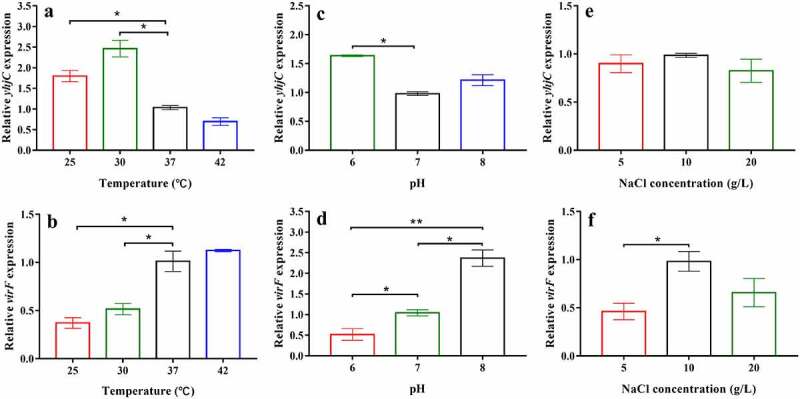
The effects of temperature, pH, and NaCl concentration on the levels of *yhjC* and *virF* mRNA. (a, b) Temperature influences the expression of *yhjC* and *virF*; (c, d) pH influences the expression of *yhjC* and *virF*; (e, f) NaCl concentrations influence the expression of *virF* but not that of *yhjC*. The relative gene expression of *Shigella* at 37 °C, pH 7, or 10 g/L NaCl were set to 1 and used as reference. Data were generated from triplicate experiments and have been presented as mean ± SD. *p*-values were determined by using unpaired Student’s *t*-test (*p < 0.05; **p < 0.01; ***p < 0.001)

The effects of temperature, pH, and NaCl concentration on the mRNA levels of *yhjC* and *virF* were investigated in the current study. The relative gene expression of *Shigella* at 37 °C, pH 7, or 10 g/L NaCl were set to 1 and used as reference. The mRNA level of *yhjC* was significantly higher at 30 °C than that observed at 37 °C (Figure 7a), although it continued to exhibit a relatively high expression at 37 °C, based on the Ct values. Consistent with the results reported by a previous study [18], *virF* mRNA level was significantly lower at 30 °C than that observed at 37 °C and 42 °C (Figure 7b). *yhjC* transcription at pH 6 was also higher than that at pH 7, whereas *virF* transcription was suppressed at pH 6 but significantly activated at pH 7 and pH 8 (Figure 7c and d). The mRNA level of *yhjC* was not significantly influenced by the NaCl concentrations studied, whereas *virF* expression was influenced by NaCl concentration (Figure 7e and f). These results indicate that *yhjC* activation can occur under permissive environmental stimuli (37 °C, pH 7) as well as under non-permissive environmental conditions. However, the *virF* transcription was not entirely relevant to the mRNA level of *yhjC* at different environmental conditions as transcription of *virF* is controlled by many various factors.

## Discussion

In this study, we investigated the function of the novel transcriptional regulator YhjC in *Shigella* pathogenicity. Thus far, only one study has reported YhjC, activating *csgD* transcription during biofilm formation in *Escherichia coli* K-12 [19]. First, we found that the ability of *Shigella* to colonize the colon of guinea pigs was significantly weakened by the deletion of *yhjC* (Figure 1). Consistent with this observation, the *yhjC* mutant was less effective at adhering to and invading Hela cells compared with the WT strain (Figure 2). Thus, we believe that YhjC is a virulence regulator responsible for *Shigella* infection and invasion, although the underlying mechanism of action is unclear. Most virulence processes associated with *Shigella* are conferred by the activities of T3SS and its secreted virulence effectors, the activation of which relies on VirF [20]. Further investigation confirmed that YhjC could bind to the promoter region of *virF* and exercises direct positive transcriptional regulation (Figures 4, 5). Therefore, we proposed that YhjC contributed to *Shigella* virulence by activating *virF* transcription. RNA sequencing results supported this theory, as all the genes controlled by *virF* including genes encoding T3SS, transcription activators of T3SS, and virulence effectors were obviously downregulated following *yhjC* deletion (Figure 6a and b). So the inability of the *yhjC* mutant to assemble a complete type III secretion apparatus and adequately synthesize virulence effectors ultimately attenuated virulence in vivo. Additionally, the reduced synthesis of translocators, chaperones, and other virulence-associated proteins also considerably attenuated *Shigella* virulence.

VirF performs transcriptional regulation in response to physical signals mediated by different regulatory factors [4]. The nucleoid-associated protein H-NS represses *virF* transcription at non-permissive temperatures (below 32 °C) by binding to the promoter region of *virF* [21,22]. Therefore, YhjC may play an important role in activating *virF* transcription, especially at temperatures below 32 °C. Perhaps this may explain why *yhjC* has a robust expression at 30 °C (Figure 7a). However, YhjC also works at the body temperature (around 37 °C), which has been confirmed by the cell and animal experiments in this study. In previous studies, phosphorylated CpxR reportedly induced direct activation of *virF* transcription by efficiently binding to its target site upstream of the *virF* gene, while CpxA repressed *virF* transcription at low pH (6.0) but activated transcription at high pH (7.4) through its action as a CpxR phosphatase [9], consistent with our results. The higher *yhjC* mRNA level observed at pH 6 might weaken CpxA repression (Figure 7c and d). Interestingly, NaCl concentration did not affect *yhjC* expression (Figure 7e). However, *virF* transcription was markedly suppressed under low osmotic conditions and was activated under physiological osmotic conditions [23], a similar phenomenon was observed in our study (Figure 7f). Considering the results of the RNA sequencing and qRT analysis in addition to these results, YhjC is indispensable to *virF* transcription, both under permissive and non-permissive environmental conditions (below 37°C, pH 6.0), although it is not the only factor that controls *virF* expression, as *virF* is extensively regulated.

Additionally, YhjC seems play much more important role in *virF* transcriptional regulation than those of Fis, IHF and CpxA/R [8–10], as expression of *virF* was downregulated by 5.59-fold following *yhjC* deletion, which was much more significant (Figure 4). These results strongly suggested that YhjC is vital additional regulator responsible for *virF* activation during *Shigella* infection. The sequence of the YhjC target binding site in the *virF* promoter region will be investigated in a future study. However, it is evident that the interaction between YhjC and the *virF* promoter is probable, due to the presence of several T-N_11_-A binding motifs in the promoter (Figure S1), consistent with the characteristics of LTTR-regulated promoters [24].

Gene expression profiling showed that YhjC was a global transcriptional regulator. Gene ontology (GO) enrichment analysis identified the following three functional categories: molecular function, cellular component, and biological process (Figure S2). Under the molecular function category, differentially expressed genes (DEGs) encoding ubiquitin-protein transferase and sequence-specific DNA-binding proteins were likely to be correlated with *Shigella* pathogenesis. Previous reports have indicated that the IpaH family proteins promote bacterial survival possess E3 ubiquitin ligase activity [25,26]. These DNA-binding proteins probably function as transcription regulators as invasion and cell-to-cell spread of *Shigella* are largely controlled by several transcription regulators, such as VirF, VirB, and MxiE [4]. GO analysis of cellular components showed that the DEG-encoded proteins were mainly located in the extracellular region, outer cell membrane, host cell cytoplasm, host cell nucleus, host cell plasma membrane, and host cell cytosol. The apparatus for T3SS of *Shigella* is located in the bacterial membrane and can be inserted into the host cell plasma membrane, while the T3SS effectors are secreted into the host cell cytoplasm. The DEGs associated with biological processes, such as pathogenesis, DNA integration, transposition, DNA recombination, protein secretion, and ion transport, have been identified, further implying that YhjC is a global regulator that can exert influence on *Shigella* virulence. Kyoto Encyclopedia of Genes and Genomes (KEGG) pathway enrichment analysis was conducted to identify pathways that were enriched in response to *yhjC* deletion (Figure S3). The most significantly enriched pathways were related to shigellosis, bacterial invasion of epithelial cells, and bacterial secretion system, indicating that YhjC functioned as a global regulator involved in *Shigella* virulence.

The *csgD* gene encoding the master regulator of biofilm formation, whose expression is controlled by YhjC, was not identified in the RNA sequencing analysis in this study, and this could be attributable to the absence of biofilm formation during the occurrence of *Shigella* virulence-induced processes [19,27]. Additional promoters that can establish interactions directly with YhjC should also be investigated. Finally, the effects of differential expression of *virF*-unrelated genes located on the large virulence plasmid and the genome exerted on bacterial virulence remain unknown and warrant further investigation.

In conclusion, we determined the virulence and global regulatory function of the novel regulator, YhjC, in *Shigella* pathogenesis. This study suggests that YhjC can activate *virF* transcription by directly binding to its promoter region. Therefore, YhjC is a critical virulence regulator necessary for promoting *Shigella* pathogenicity. YhjC expression may also control *Shigella* growth and survival through certain unidentified mechanisms.

## Materials and methods

### Bacterial strains and plasmids

The *Shigella flexneri* 5a strain M90T was used as the wild-type strain in this study. The *yhjC* mutant was generated using the λ Red recombinase system, supported by the pSim17 plasmid (blastidin-resistant) encoding three proteins (Exo, Beta, and Gam) required for homologous recombination [28]. First, the M90T strain harboring the pSim17 plasmid was established through electrotransformation. Then, DNA fragments composed sequentially (5ʹ→3ʹ) of an upstream 50-bp sequence of the target gene, the chloramphenicol-resistant gene sequence, and the downstream 50-bp reverse complementary sequence of the target gene were subjected to PCR amplification. Chloramphenicol-resistant pKD3 plasmid was used as the template and primers were designed to cover the 50-bp homologous arm sequences. Next, the DNA fragments were introduced into competent M90T cells using electrotransformation. Finally, the cells were recovered at 37°C for 2 h and suspensions of recovered cells were spread on agar plates containing 25 μg/mL chloramphenicol to obtain single mutant colony. To validate the formation of the *yhjC* mutant strain, the *yhjC* loci in the chloramphenicol-resistant colony were amplified and preliminarily identified through electrophoresis, and further identification was performed through Sanger sequencing.

The *yhjC* gene and its upstream 500-bp sequence were cloned into the plasmid pBR322 at a region between *NheI* and *BamHI* restriction enzyme digestion sites, and the resulting constructs were electrotransformed into competent *yhjC* mutants; subsequently, after screening the bacteria for resistance to chloramphenicol and ampicillin on agar plates containing the two antibiotics, the complemented strain (C*yhjC*) was constructed. To perform expression and purification of the YhjC-His6 protein, the pET-*yhjC* plasmid was established by cloning the *yhjC* gene sequence into the pET-28a plasmid between *BamHI* and *XhoI* sites downstream of the His-tag. All constructed plasmids and strains were validated using the methods described in the identification of *yhjC* mutants. Primers used in this study are listed in Table S1.

### Growth conditions for bacteria and Hela cells

Bacteria were cultured in either liquid LB broth (1% tryptone, 0.5% yeast extract, and 1% NaCl) or solid LB medium (liquid LB broth supplemented with 1.5% agar) at 37°C. Where necessary, antibiotics were added at the following final concentrations: 25 μg/mL chloramphenicol, 50 μg/mL kanamycin and 100 μg/mL ampicillin. HeLa cells used in this study were cultured in the Dulbecco’s modified Eagle medium (DMEM) with 10% fetal bovine serum at 37°C under 5% CO_2_. Next, 24 h before infection with bacteria, HeLa cells were dissociated using trypsin, and the cells (1 × 10^5^ cells/well) were seeded into 12-well culture plates to achieve final differentiated cell monolayers.

### Intrarectal infection of guinea pigs

Three-week-old female Dunkin-Hartley guinea pigs (< 150 g) were used for conducting the intrarectal infection experiment [29]. To screen for *Shigella* colonization in the colons, the WT bacterial strain was transformed with the pBR322 plasmid, and the transformation conferred the ability to grow on agar plates containing ampicillin; the *yhjC* and complemented strains harbor genes for chloramphenicol and ampicillin resistance, respectively. *Shigella* was first cultured in LB medium at 37°C for approximately 13 h, and then sub-cultured in LB medium at an inoculation ratio of 1:100. When the OD_600_ of the bacterial culture reached a value of 0.6 (indicating logarithmic phase of the bacteria), the bacterial cells were collected by centrifugation and were suspended in PBS. Guinea pigs were subjected to fasting conditions 15 h prior to intrarectal infection, 5 guinea pigs per group were used. 10^10^ *Shigella* CFUs (100 μL) were injected into the rectum of guinea pigs anesthetized with diethyl ether. After an incubation period of 8 h, the guinea pigs were euthanized, and the distal 5-cm specimens of the colon were harvested. Each colon specimen was ground, and bacterial CFUs were counted by conducting surface spread of bacterial suspensions on agar plates containing the corresponding antibiotics.

### Adhesion and invasion assays

Adhesion and invasion assays were performed as per methods previously described with slight modifications [5,30]. Briefly, overnight *Shigella* cultures were sub-cultured into fresh LB medium at an inoculation ratio of 1:100 and grown at 37°C to achieve an OD_600_ of 0.6. The bacterial cells were then pelleted by centrifugation, and the supernatant was discarded. The retrieved pellets were suspended in fresh DMEM for 20 min. HeLa cells were infected with *Shigella* cells at a multiplicity of infection (MOI) of 100:1 (ration of the number of bacterial cells to HeLa cells), and the bacteria-HeLa cell mixed suspension was centrifuged at 800 × g for 5 min. Then, HeLa cells and bacterial cells were co-cultured at 37°C to initiate invasion. For adhesion assays, host cells were subjected to washing steps three times using sterile PBS 40 min post-infection to remove bacterial cells that did not adhere to the host cells. Subsequently, the cells were subjected to lysis using 0.1% Triton X-100, and the bacterial cells that could invade HeLa cells were enumerated using the flat colony counting method. The adhesion rate was calculated by dividing the number of recovered bacteria by the total number of bacterial cells used for infection.

For conducting invasion assays, 100 μg/mL gentamicin was added to the cell culture medium 40 min post-infection, and the cells were incubated for 1 h to kill the extracellular bacteria. 100 min post infection, HeLa cells were subjected to washing steps and lysed using 0.1% Triton X-100 to release the intracellular bacterial cells, and the lysates were spread on agar plates for enumeration of bacterial CFUs. The invasion rate was calculated as the ratio of the number of recovered bacteria to the total number of bacterial cells used for infection.

### Congo red binding assay

The Congo red binding assay was performed as per protocols previously published [12]. The WT strains and *yhjC* mutants were cultured overnight in LB broth. The next day, the cultures were serially diluted, and 100 μL of each dilution was added onto trypticase soy broth (TSB) agar plates supplemented with 0.01% (w/v) Congo red (hereafter referred to as Congo red plates). The plates were incubated overnight at 37°C. A density of approximately 6 × 10^9^ cells/mL was used to quantify the relative amount of Congo red bound by the bacterial cells. Ten culture spots for each sample were scraped off the agar plate and suspended in 750 μL 25% ethanol to remove Congo red bound to the cells. The optical density of the cell suspension was measured at 600 nm (OD_600_) to perform normalization of the samples to the cell number. Cell suspensions were then centrifuged, and the optical density of the supernatant was measured at 498 nm (OD_498_) to quantify the amount of Congo red released from the bacteria. Relative Congo red binding was determined as: OD_498_/OD_600_.

### RNA preparation

*Shigella* cells were cultured overnight in LB at 37°C and sub-cultured at an inoculation ratio of 1:100. When the optical density at 600 nm (OD_600_) reached a value of 0.6, indicating the logarithmic phase of the bacteria, *Shigella* cells were collected by centrifugation at 5000 rpm for 5 min and were resuspended in sterile PBS. The bacterial cells were then incubated in 50 µg/mL Congo red solution at 37°C for 20 min to induce T3SS activity in *Shigella* in vitro [31]. Total RNA extraction was performed using the TRIzol reagent (Invitrogen, USA) according to the manufacturer’s instructions. The RNA samples were purified using the RNeasy Mini Kit and were treated with DNase I (QIAGEN, Germany) to remove traces of contaminating DNA. The final RNA concentration was determined using the NanoDrop 2000 spectrophotometer (NanoDrop, USA).

### qRT-PCR analysis

qRT-PCR was performed using the Applied Biosystems 7500 Real-Time PCR system (Applied Biosystems, USA). The primers used for the qRT-PCR analysis are listed in Table S1. To analyze the expression of virulence genes, RNA samples from the bacteria (collected in the logarithmic phase) were prepared as per methods described in Section 4.6. A total of 1.2 μg RNA from each sample was reverse-transcribed into cDNA using the PrimeScript^TM^ RT Reagent kit (TaKaRa, Japan). The total qRT-PCR reaction mixture (20 μL) contained 10 μL of the PowerUp™ SYBR™ Green Master Mix (Applied Biosystems, USA), 1 μL of cDNA (approximately 40 ng), 1 μL of forward primer, and 1 μL of reverse primer, each at a final concentration of 0.5 μM. The data were normalized using the 16S rRNA gene as a reference control. Target gene expression levels were calculated using the 2^−ΔΔCt^ method. Each qRT-PCR experiment was performed in triplicate.

### Library construction and RNA sequencing

RNA sequencing was performed using the Illumina sequencing platform. The NEBNext Ultra Directional RNA Library Prep Kit (NEB, USA) was used for cDNA library construction following the manufacturer’s instructions. Reads obtained from the sequencing experiment were mapped to the reference genomes of *S. flexneri* 5a M90T and its large virulence plasmid pWR100. HTSeq (version 0.6.1) was used to count the number of reads. Gene expression levels were calculated using the FPKM method, where FPKM represents fragments per kilobase of exon model per million reads mapped [32]. The DESeq2 (v1.6.3) package was used to compare differences in gene expression between the WT and Δ*yhjC* strains. Differentially expressed genes were those with |fold-change| ≥ 2 and *p* < 0.05 [33].

### Electrophoretic mobility shift assay

The pET28a-*yhjC* plasmid was transformed into *Escherichia coli* BL21 (DE3), and YhjC harboring a C-terminal 6× His tag (YhjC-His_6_ protein) was expressed after subjection to induction using isopropylthio-β-galactoside (IPTG). The protein was purified from the lysate supernatant of the DE3 using a HiTrap Ni^2+^-chelating column (GE Healthcare, Germany), as per methods described previously [34]. Protein concentration was determined according to the Bradford method and the samples were stored at −80°C.

EMSA was performed as previously described with some modifications [35]. Briefly, 300-bp DNA fragments of the *virF* and *rpoS* promoter regions were amplified by conducting PCR and were subjected to purification steps. A quantity of 50 ng of the promoter fragments and different concentrations (0–50 nM) of the purified YhjC protein were incubated at 26°C for 30 min in a 20-μL solution containing EMSA binding buffer (20 mM Tris HCl pH 7.5, 50 mM KCl, 1 mM EDTA, 1 mM dithiothreitol, and 5% glycerol). Samples were then loaded onto a 6% polyacrylamide gel immersed in 0.5 × Tris-Borate-EDTA for electrophoresis. DNA fragments were stained with Gel Red (Biotium, USA).

### Statistical analysis

Data were analyzed using GraphPad Prism (version 7.00; La Jolla, CA, USA). The data presented in each figure or table represent mean ± standard deviation (SD) values obtained from the conduction of three independent experiments. Student’s *t*-test was used to analyze significant differences between the two groups. Differences were considered significant at *p* < 0.05 (*p < 0.05; **p < 0.01; ***p < 0.001).

## Supplementary Material

Supplemental Material

## Data Availability

The RNA sequencing data acquired in this study are available in the NCBI Sequence Read Archive (SRA, PRJNA721543).
