# Supplementary material for: YhjC is a novel transcriptional regulator required for Shigella flexneri virulence
Source: Virulence. 2021 Jun 21;12(1):1661–71. doi: 10.1080/21505594.2021.1936767 (PMC8218686; doi:10.1080/21505594.2021.1936767)
Supplement: Supplemental Material [file KVIR_A_1936767_SM1984.zip › supplementary/Supplementary Materials_revised-5.21clean.docx]

Table S1 Primers used in this study

| Targets | Primer sequences (5'-3') |
| --- | --- |
| Primers for qRT-PCR analysis | |
| *16S rRNA* | CTGAGAGGATGACCAGCCACACT |
|  | GGTGCTTCTTCTGCGGGTAACG |
| *virF* | TTCAATGACGGTTAGCTCAGGCAAT |
|  | TCGATAGAAACCTCCTCCTCAGAGA |
| *virB* | GCATCCGAGAACTTGGTATTGGTCT |
|  | GTGGAACGCTTGCTGCCTGAA |
| *ipaA* | AGGCAACTTCACCATCATCAACAGA |
|  | TGGCATACGCATTCATTGAGTTGGA |
| *ipaB* | GTCCTAGTTGCAGCAGTCGTTCTC |
|  | TCCCGCTTGTGTGGCAGAGTTA |
| *ipaC* | ACCTTGCCACTGCTCAATCTCTTG |
|  | GCTCGTAAGTTCTTCTCTGGGTGTC |
| *MxiE* | GTGTCTTTGAAGCAGGGAGAGAGG |
|  | GCACACCCACTCAGTGTTAATAGCA |
| *ushA* | GTTATTGGGCTGACAACCGATGACA |
|  | TTGCGAGTGACCACCGACGAT |
| *fucO* | TGCGGCAGAAGTGACCATTAACTAC |
|  | TCCTTATCACCAGCAACCGATCCT |
| *nirC* | GGTATCGCCTTAACGCTGGTGATT |
|  | ACGGAGTGAACGATGCTGGTATCTA |
| *carB* | TGAAGAAGAGTGCGAAGCGAATCC |
|  | ATCGGAAGTGTCGTAGTCGGTAGAG |
| *trpE* | GATGAAGAGTTCGGTGGCGTAGTG |
|  | TGGCGGCTGGTGGCATCATA |
| *ylcB* | GCAGCAGTCGGTGGTGAATTATGA |
|  | TCGGCATCCAGCACTTCCAGATA |
| Primers for *yhjC* mutant establishment | |
|  | CTGAGAAATCGCCACATTCGTCATGACAACATTGTGAAACCCGGCATTAGGTGTAGGCTGGAGCTGCTTC |
|  | ATCCGCTCTTTTGTCCTTCTGTCGTGGTCATTCTTAAAAAGTATAGTCGGCATATGAATATCCTCCTTAG |
| Primers for pBR322-*yhjC* plasmid construction | |
|  | CTAGCTAGCATTTTTATTCCATGAATAAATATTGACTGCCTGTCAAACA |
|  | CGGGATCCTCAGTCCAGGTGCTCTTTCATTACG |
| Primers for pET28a-*yhjC* plasmid construction | |
|  | CGCGGATCCATGGATAAAATTCACGCAAT |
|  | CCCCTCGAGGTCCAGGTGCTCTTTCATTA |
| Primers for amplification of the promoter region of *virF* | |
|  | GTTATGCAAGCGAACCTTT |
|  | GGTTATAGTCCCTTTCAGTG |

Table S2 Expression profile of downregulated genes in Δ*yhjC*

| Gene name | log_2_ (Δ*yhjC*/WT) | *p*-value | Product |
| --- | --- | --- | --- |
| *CP0215* | -6.826 | 2.190E-08 | hypothetical protein |
| *CP0088* | -6.046 | 2.880E-06 | IS629 ORF1 |
| *CP0062* | -5.862 | 7.860E-06 | ISSfl1 ORF2 |
| *CP0072* | -5.675 | 4.179E-04 | IS1294 transposase |
| *CP0030* | -5.609 | 6.770E-17 | iso-IS1 ORF2 |
| *CP0011* | -5.451 | 1.099E-04 | hypothetical protein |
| *CP0086* | -5.398 | 2.500E-20 | hypothetical protein |
| *insB* | -5.385 | 2.280E-12 | IS1 ORF2 |
| *CP0073* | -5.324 | 1.250E-11 | ISSfl1 ORF1 |
| *CP0043* | -5.247 | 3.121E-04 | IS1294 transposase |
| *CP0068* | -5.156 | 1.180E-33 | hypothetical protein |
| *acp* | -5.154 | 7.970E-80 | putative acyl carrier protein |
| *CP0021* | -5.123 | 0.000E+00 | transposase |
| *yjbG* | -5.113 | 4.644E-03 | hypothetical protein |
| *CP0069* | -4.854 | 1.070E-163 | IS21 ORF2 |
| *CP0044* | -4.809 | 3.280E-19 | IS150 ORF1(ORF A) |
| *CP0075* | -4.761 | 9.392E-03 | IS630 ORF |
| *CP0077* | -4.754 | 1.450E-11 | iso-IS1 ORF2 |
| *CP0060a* | -4.733 | 2.550E-06 | hypothetical protein |
| *CP0076* | -4.661 | 6.100E-26 | iso-IS1 ORF1 |
| *icsP/sopA* | -4.570 | 0.000E+00 | outer membrane protease |
| *CP0214* | -4.551 | 7.930E-12 | IS100 ORF2 |
| *CP0087* | -4.511 | 1.450E-26 | IS629 ORF2 |
| *CP0014* | -4.477 | 4.790E-237 | hypothetical protein |
| *SF2441* | -4.299 | 8.130E-09 | formyl-coenzyme A transferase |
| *CP0047* | -4.289 | 2.050E-109 | IS2 ORF2 |
| *CP0041* | -4.176 | 4.500E-07 | IS150 ORF B |
| *sepA* | -4.165 | 0.000E+00 | extracellular serine protease SepA |
| *phoN2/apy* | -4.115 | 1.340E-298 | periplasmic phosphatase; apyrase; ATP diphosphohydrolase |
| *CP0071* | -4.095 | 3.980E-14 | IS1294 transposase |
| *parB* | -4.095 | 8.120E-227 | plasmid segregation protein |
| *CP0270* | -4.065 | 1.660E-25 | IS3 ORF2 |
| *parA* | -4.022 | 6.580E-275 | plasmid segregation protein |
| *CP0033* | -4.004 | 1.850E-40 | transposase |
| *ipgH* | -4.002 | 3.000E-79 | invasion plasmid gene product |
| *CP0012* | -3.971 | 1.340E-225 | hypothetical protein |
| *CP0059* | -3.952 | 3.120E-125 | hypothetical protein |
| *CP0037* | -3.903 | 2.422E-04 | hypothetical protein |
| *CP0023* | -3.895 | 3.650E-62 | hypothetical protein |
| *CP0060* | -3.792 | 1.990E-31 | hypothetical protein |
| *insA* | -3.737 | 4.810E-45 | IS1 ORF1 |
| *CP0168* | -3.731 | 2.020E-140 | hypothetical protein |
| *ipaH2.5* | -3.731 | 1.960E-05 | invasion plasmid antigen |
| *CP0116* | -3.676 | 7.080E-92 | IS600 ORF1 |
| *CP0039* | -3.636 | 1.290E-24 | IS629 ORF2 |
| *CP0180* | -3.278 | 7.000E-101 | IS629 ORF1 |
| *CP0056* | -3.145 | 1.000E-39 | IS629 ORF2 |
| *SF2440* | -3.142 | 7.460E-07 | oxalyl-CoA decarboxylase |
| *CP0117* | -3.110 | 3.710E-101 | IS600 ORF2 |
| *emrK* | -3.065 | 4.276E-03 | multidrug resistance protein K |
| *SF1538* | -3.030 | 5.470E-08 | oxidoreductase |
| *CP0025* | -2.964 | 1.090E-73 | IS3 ORF2 |
| *CP0100* | -2.929 | 1.910E-12 | hypothetical protein |
| *SF1537* | -2.880 | 1.016E-04 | transcriptional regulator YdeO |
| *CP0002* | -2.799 | 4.280E-20 | resolvase |
| *CP0269* | -2.754 | 7.932E-03 | IS911 ORF2 |
| *emrY* | -2.682 | 2.368E-04 | multidrug resistance protein Y |
| *CP0102* | -2.552 | 2.030E-10 | hypothetical protein |
| *CP0057* | -2.531 | 5.650E-18 | IS600 ORF2 |
| *CP0099* | -2.476 | 1.050E-10 | ISSfl1 ORF1 |
| *ipaH_4* | -2.438 | 2.140E-23 | invasion plasmid antigen |
| *CP0095* | -2.431 | 1.839E-03 | ISSfl4 ORF3 |
| *yfdV* | -2.376 | 1.077E-02 | transporter YfdV |
| *icsA*/*virG* | -2.350 | 2.950E-176 | hypothetical protein |
| *yrfB* | -2.283 | 2.962E-03 | hypothetical protein |
| *CP0186* | -2.164 | 9.600E-23 | hypothetical protein |
| *ipaH_3* | -2.140 | 1.070E-17 | invasion plasmid antigen |
| *SF0364* | -2.131 | 1.370E-05 | hypothetical protein |
| *SF1010* | -2.115 | 1.432E-03 | hypothetical protein |
| *SF2423* | -2.031 | 2.490E-13 | hypothetical protein |
| *ipaH_1* | -2.029 | 3.990E-80 | invasion plasmid antigen |
| *CP0167* | -1.981 | 1.830E-16 | hypothetical protein |
| *CP0267* | -1.962 | 9.820E-08 | IS600 ORF1 |
| *CP0184* | -1.907 | 2.270E-46 | IS629 ORF2 |
| *ushA* | -1.762 | 6.320E-82 | bifunctional UDP-sugar hydrolase/5'-nucleotidase |
| *CP0179* | -1.754 | 5.530E-59 | IS629 ORF2 |
| *CP0118* | -1.707 | 7.690E-10 | IS150 ORF B |
| *CP0007* | -1.686 | 3.229E-03 | IS600 ORF2 |
| *CP0016* | -1.654 | 4.340E-08 | IS91 ORF2 |
| *ymcB* | -1.651 | 8.535E-04 | hypothetical protein |
| *yjiY* | -1.616 | 3.250E-79 | carbon starvation protein |
| *SF2442* | -1.564 | 6.744E-03 | hypothetical protein |
| *CP0158* | -1.523 | 5.150E-05 | IS600 ORF1 |
| *CP0157* | -1.476 | 2.640E-21 | IS600 ORF2 |
| *yhfT* | -1.442 | 6.606E-04 | transport system permease |
| *nirC* | -1.395 | 1.799E-04 | nitrite reductase subunit NirC |
| *fucO* | -1.385 | 5.250E-30 | L-2-propanediol oxidoreductase |
| *cpsB* | -1.382 | 3.639E-03 | mannose-1-phosphate guanylyltransferase |
| *lpfA* | -1.373 | 5.104E-03 | fimbrial protein |
| *SF2505* | -1.354 | 5.920E-45 | bifunctional malic enzyme oxidoreductase/phosphotransacetylase |
| *CP0178* | -1.333 | 9.654E-03 | IS3 ORF2 |
| *SF1699* | -1.313 | 1.455E-03 | oxidoreductase%2C Fe-S subunit |
| *cstA* | -1.297 | 4.440E-31 | carbon starvation protein A |
| *dctA* | -1.284 | 9.330E-50 | C4-dicarboxylate transporter DctA |
| *soxS* | -1.276 | 1.690E-16 | DNA-binding transcriptional regulator SoxS |
| *celF* | -1.266 | 1.632E-04 | 6-phospho-beta-glucosidase |
| *CP0174* | -1.263 | 4.666E-03 | transposase |
| *glpC* | -1.198 | 1.440E-09 | sn-glycerol-3-phosphate dehydrogenase subunit C |
| *SF3510* | -1.180 | 2.220E-06 | hypothetical protein |
| *CP0239* | -1.178 | 2.231E-03 | IS630 ORF |
| *sdhA* | -1.156 | 7.180E-47 | succinate dehydrogenase flavoprotein subunit |
| *lldP* | -1.155 | 4.680E-26 | L-lactate permease |
| *dadA* | -1.153 | 1.670E-25 | D-amino acid dehydrogenase small subunit |
| *acpS* | -1.141 | 4.512E-03 | 4'-phosphopantetheinyl transferase |
| *CP0026* | -1.119 | 2.030E-05 | IS100 ORF1 |
| *iadA* | -1.111 | 7.900E-16 | isoaspartyl dipeptidase |
| *yecP* | -1.099 | 2.730E-09 | tRNA mo(5)U34 methyltransferase |
| *ybjH* | -1.080 | 7.755E-03 | hypothetical protein |
| *yjiX* | -1.075 | 4.320E-05 | hypothetical protein |
| *CP0112* | -1.056 | 1.641E-04 | ISSfl4 ORF3 |
| *yhiH* | -1.048 | 5.010E-32 | ABC transporter ATP-binding protein |
| *nhaR* | -1.045 | 3.210E-13 | transcriptional activator NhaR |
| *eutA* | -1.032 | 1.010E-02 | reactivating factor for ethanolamine ammonia lyase |
| *SF1741* | -1.027 | 2.920E-08 | ABC transporter ATP-binding protein |
| *CP0020* | -1.017 | 1.150E-33 | ISSfl4 ORF3 |
| *yjiA* | -1.014 | 1.190E-13 | GTP-binding protein YjiA |

Note: T3SS-related genes have been shown in Figure 1. Gene name were referenced from the *S*. *flexneri* serotype 2a strain 301 and exist as well as conserve in *S. flexneri* serotype 5a strain M90T.

Table S3 Expression profile of upregulated genes in Δ*yhjC*

| Gene name | log_2_ (Δ*yhjC*/WT) | *p*-value | Product |
| --- | --- | --- | --- |
| *uraA* | 1.011 | 1.725E-03 | uracil transporter |
| *gidB* | 1.012 | 4.769E-04 | 16S rRNA methyltransferase GidB |
| *SF3561a* | 1.019 | 3.168E-03 | endonuclease III |
| *rfbI* | 1.022 | 3.530E-05 | glycosyl translocase |
| *fhuF* | 1.027 | 1.510E-05 | ferric hydroximate transport ferric iron reductase |
| *yhaM* | 1.040 | 2.140E-16 | hypothetical protein |
| *ycaD* | 1.046 | 2.737E-04 | MFS family transporter protein |
| *rpsS* | 1.051 | 5.590E-20 | 30S ribosomal protein S19 |
| *yaaH* | 1.053 | 1.050E-06 | hypothetical protein |
| *cytR* | 1.060 | 7.310E-06 | DNA-binding transcriptional regulator CytR |
| *rplV* | 1.061 | 5.520E-14 | 50S ribosomal protein L22 |
| *ybaQ* | 1.074 | 2.430E-18 | hypothetical protein |
| *acrR* | 1.079 | 5.581E-03 | DNA-binding transcriptional repressor AcrR |
| *groES* | 1.083 | 1.420E-34 | co-chaperonin GroES |
| *yhdG* | 1.110 | 3.140E-18 | dehydrogenase |
| *SF1896* | 1.112 | 1.769E-03 | DNA-packaging protein |
| *glpA* | 1.112 | 1.600E-12 | sn-glycerol-3-phosphate dehydrogenase subunit A |
| *tdcB* | 1.135 | 2.590E-08 | threonine dehydratase |
| *SF3768* | 1.141 | 5.870E-08 | hypothetical protein |
| *yjfM* | 1.145 | 6.264E-04 | hypothetical protein |
| *entE* | 1.168 | 1.260E-05 | 2,3-dihydroxybenzoate-AMP ligase |
| *SF1620* | 1.195 | 1.484E-04 | multidrug efflux system protein MdtI |
| *yhcL* | 1.197 | 9.323E-04 | cryptic C4-dicarboxylate transporter DcuD |
| *bglJ* | 1.202 | 2.926E-03 | 2-component transcriptional regulator |
| *SF2921* | 1.210 | 4.930E-06 | oxidoreductase |
| *ibpA* | 1.227 | 1.950E-17 | heat shock protein IbpA |
| *rplC* | 1.228 | 1.910E-42 | 50S ribosomal protein L3 |
| *yfbM* | 1.233 | 7.286E-04 | hypothetical protein |
| *gntK* | 1.251 | 3.550E-20 | gluconokinase |
| *entF* | 1.256 | 4.330E-14 | enterobactin synthase subunit F |
| *ychM* | 1.257 | 3.150E-06 | transporter |
| *yheA* | 1.265 | 1.650E-06 | bacterioferritin-associated ferredoxin |
| *slp* | 1.283 | 1.560E-50 | carbon starvation outer membrane protein |
| *SF1356* | 1.289 | 1.349E-03 | insertion sequence element IS600 transposase |
| *SF2038* | 1.299 | 7.412E-03 | lysis protein S |
| *entD* | 1.305 | 3.201E-03 | enterobactin synthase multienzyme complex phosphopantetheinyltransferase |
| *hdeD* | 1.316 | 6.660E-53 | acid-resistance membrane protein |
| *dmsB* | 1.318 | 1.542E-03 | anaerobic dimethyl sulfoxide reductase subunit B |
| *cysU* | 1.326 | 1.460E-09 | sulfate/thiosulfate transporter subunit |
| *rpsJ* | 1.339 | 3.190E-36 | 30S ribosomal protein S10 |
| *SF1631* | 1.343 | 1.180E-05 | L-cystine transporter TcyP |
| *insA* | 1.349 | 3.147E-03 | insertion element IS1 protein InsA |
| *fepA* | 1.355 | 3.170E-14 | outer membrane receptor FepA |
| *fliZ* | 1.381 | 6.516E-04 | flagella biosynthesis protein FliZ |
| *yegB* | 1.388 | 1.413E-03 | transporter |
| *carB* | 1.400 | 1.960E-32 | carbamoyl phosphate synthase large subunit |
| *fhuD* | 1.402 | 1.490E-06 | iron-hydroxamate ABC transporter substrate-binding protein |
| *fes* | 1.417 | 8.890E-06 | enterobactin/ferric enterobactin esterase |
| *adiY* | 1.418 | 8.370E-15 | AraC family transcriptional regulator |
| *bioF* | 1.423 | 8.352E-03 | 8-amino-7-oxononanoate synthase |
| *fepC* | 1.430 | 7.940E-06 | iron-enterobactin ABC transporter ATP-binding protein |
| *SF1621* | 1.432 | 6.810E-09 | multidrug efflux system protein MdtJ |
| *fhuA* | 1.467 | 5.470E-30 | ferrichrome outer membrane transporter |
| *SF4401* | 1.469 | 8.138E-03 | hypothetical protein |
| *yhaO* | 1.509 | 2.680E-16 | transport system permease |
| *SF1755* | 1.521 | 6.597E-03 | porin |
| *fhiA* | 1.524 | 4.570E-06 | flagellar biosynthetic protein FlhA |
| *ycfR* | 1.551 | 1.572E-03 | hypothetical protein |
| *fhuC* | 1.578 | 3.680E-07 | iron-hydroxamate ABC transporter ATP-binding protein |
| *SF0240* | 1.589 | 2.580E-03 | hypothetical protein |
| *tdcA* | 1.591 | 1.300E-16 | DNA-binding transcriptional activator TdcA |
| *hdeB* | 1.595 | 3.770E-60 | acid-resistance protein |
| *SF1525a* | 1.612 | 2.787E-03 | hypothetical protein |
| *SF1649a* | 1.616 | 6.148E-03 | beta-lactam resistance family protein |
| *hdeA* | 1.628 | 2.410E-81 | acid stress chaperone HdeA |
| *fepD* | 1.639 | 7.120E-10 | iron-enterobactin ABC transporter permease |
| *cysC* | 1.666 | 3.308E-03 | adenylyl-sulfate kinase |
| *phoE* | 1.676 | 4.720E-08 | outer membrane phosphoporin protein E |
| *SF1772* | 1.727 | 7.349E-03 | hypothetical protein |
| *SF0867* | 1.731 | 1.369E-04 | bacteriophage protein |
| *mtr* | 1.751 | 2.430E-14 | tryptophan permease |
| *insA* | 1.759 | 7.462E-03 | insertion element IS1 protein InsA |
| *SF3623* | 1.782 | 3.402E-03 | L-dehydroascorbate transporter large permease |
| *pyrB* | 1.790 | 1.530E-40 | aspartate carbamoyltransferase |
| *SF4488* | 1.842 | 1.850E-08 | tRNA-Ala |
| *ydhC* | 1.855 | 1.590E-17 | transporter |
| *insA* | 1.913 | 4.530E-06 | insertion element IS1 protein InsA |
| *yhiE* | 1.941 | 1.000E-121 | hypothetical protein |
| *insB* | 1.959 | 1.024E-02 | insertion element IS1 protein InsB |
| *fepG* | 2.006 | 2.020E-08 | iron-enterobactin ABC transporter permease |
| *SF4551* | 2.008 | 6.373E-03 | tRNA-Leu |
| *flhC* | 2.028 | 1.880E-12 | transcriptional activator FlhC |
| *pyrI* | 2.077 | 1.110E-27 | aspartate carbamoyltransferase |
| *SF4468* | 2.083 | 2.469E-03 | tRNA-Met |
| *SF0864* | 2.124 | 5.560E-05 | bacteriophage protein |
| *shiE* | 2.154 | 5.510E-03 | hypothetical protein |
| *yhjD* | 2.251 | 2.150E-164 | hypothetical protein |
| *ybdA* | 2.295 | 1.700E-11 | enterobactin exporter EntS |
| *SF1574* | 2.377 | 1.646E-03 | hypothetical protein |
| *ylcB* | 2.849 | 9.880E-05 | copper/silver efflux system outer membrane protein CusC |
| *SF3017a* | 2.864 | 1.390E-03 | glycolate oxidase FAD binding subunit |
| *insB* | 2.970 | 2.120E-06 | insertion element IS1 protein InsB |
| *nrdF* | 3.067 | 5.046E-04 | ribonucleotide-diphosphate reductase subunit beta |
| *trpE* | 3.168 | 2.300E-21 | anthranilate synthase component I |
| *yhjB* | 3.503 | 1.800E-45 | regulator |
| *SF1546* | 4.267 | 4.560E-06 | bacteriophage protein |
| *SF2453* | 4.862 | 7.608E-03 | PTS system transporter subunit IIB |
| *rrfG* | 5.172 | 6.376E-04 | 5S ribosomal RNA |
| *insA* | 5.384 | 1.373E-03 | insertion element IS1 protein InsA |

Note: Gene name were referenced from the *S*. *flexneri* serotype 2a strain 301 and exist as well as conserve in *S. flexneri* serotype 5a strain M90T.

**Figure legends in Supplementary Material**

Figure S1. Analysis of the *virF* promoter region. “-35” to “-10” highlighted in green indicate the RNA polymerase binding regions. “+1” represents the transcriptional start site. Sequences denoted by using a shadow mark represent the T-N_11_-A motifs.

Figure S2. GO enrichment analysis of differentially expressed genes (DEGs) between the wild type (WT) and Δ*yhjC* strains of *Shigella*. Data were generated from triplicate experiments. Only the DEGs with | log_2_ (fold-change) | ≥ 1 have been presented.

Figure S3. KEGG pathway enrichment analysis of differentially expressed genes (DEGs) between the wild type (WT) and Δ*yhjC* strains of *Shigella*. Data were generated from triplicate experiments. Only DEGs with | log_2_ (fold-change) | ≥ 1 have been presented.
