# Supplementary figures and images for: YhjC is a novel transcriptional regulator required for Shigella flexneri virulence
Source: Virulence. 2021 Jun 21;12(1):1661–71. doi: 10.1080/21505594.2021.1936767 (PMC8218686; doi:10.1080/21505594.2021.1936767)

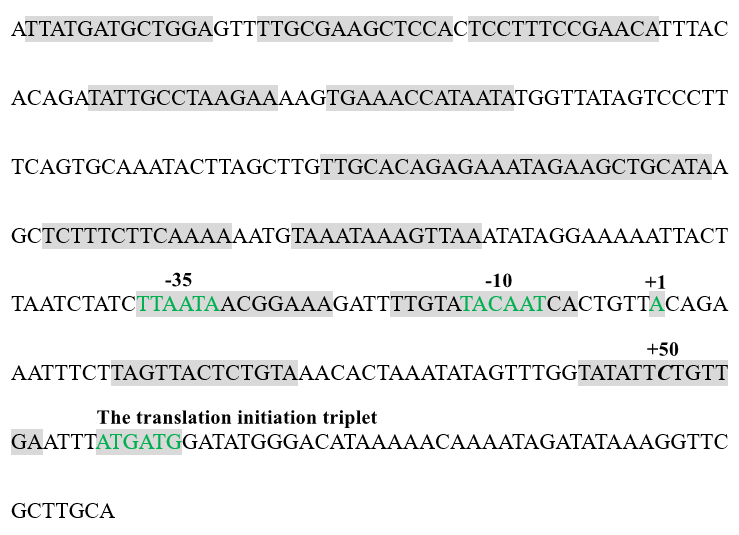

Supplement: Supplemental Material [file KVIR_A_1936767_SM1984.zip › supplementary/Figure S1.tiff]

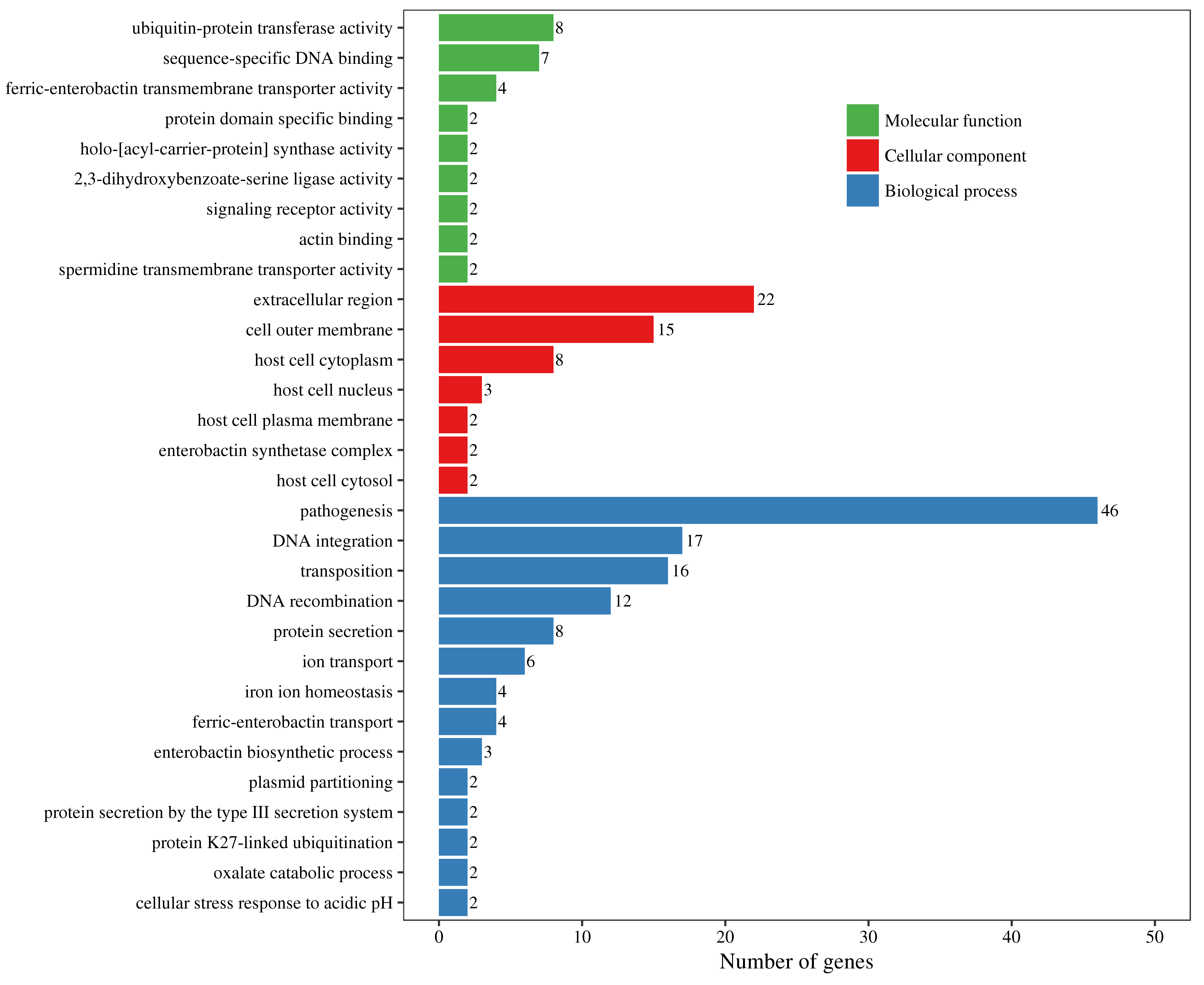

Supplement: Supplemental Material [file KVIR_A_1936767_SM1984.zip › supplementary/Figure S2.tif]

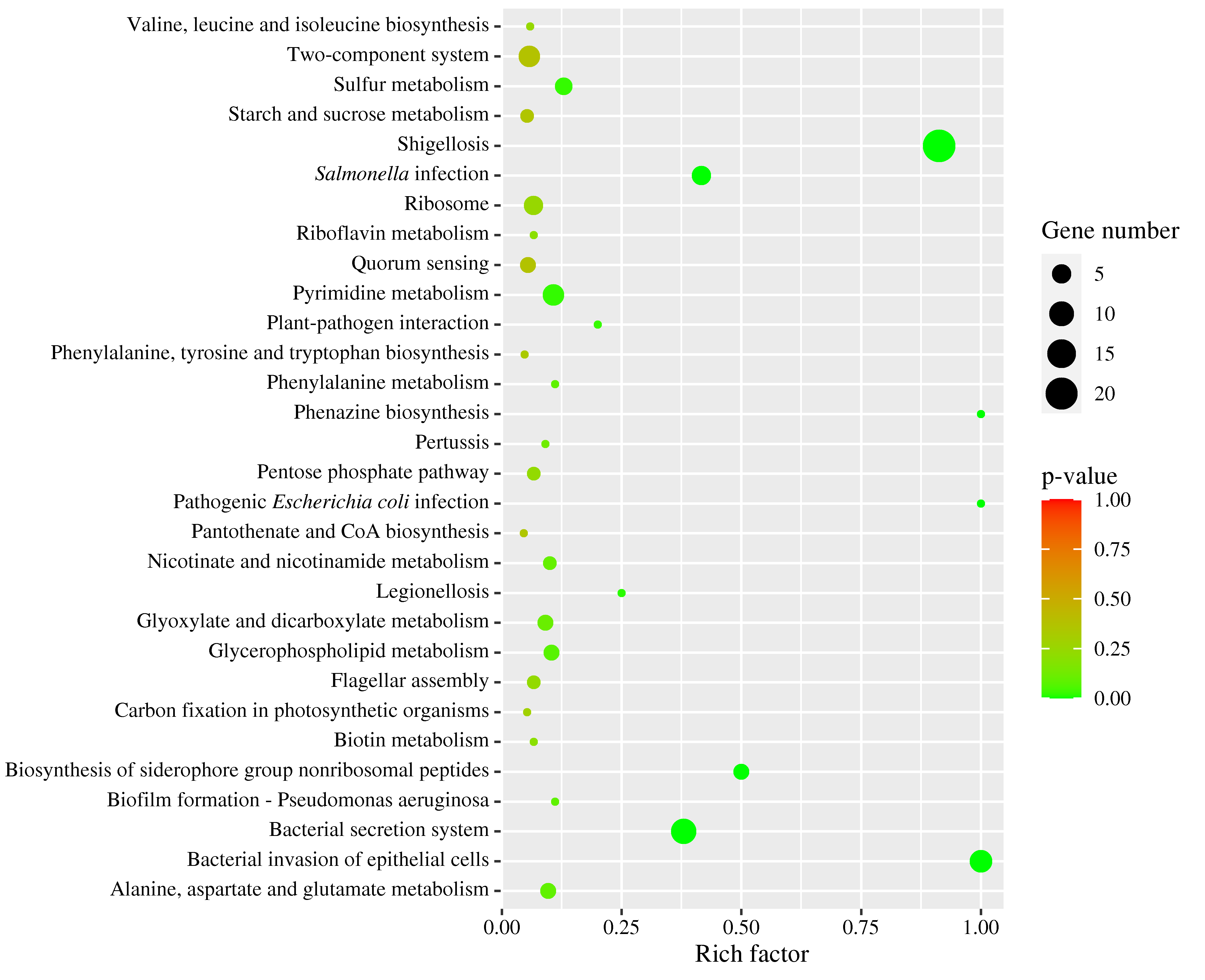

Supplement: Supplemental Material [file KVIR_A_1936767_SM1984.zip › supplementary/Figure S3.tif]
